# Early observed transient prostate-specific antigen elevations on a pilot study of external beam radiation therapy and fractionated MRI guided High Dose Rate brachytherapy boost

**DOI:** 10.1186/1748-717X-1-28

**Published:** 2006-08-16

**Authors:** Anurag K Singh, Peter Guion, Robert C Susil, Deborah E Citrin, Holly Ning, Robert W Miller, Karen Ullman, Sharon Smith, Nancy Sears Crouse, Denise J Godette, Bronwyn R Stall, C Norman Coleman, Kevin Camphausen, Cynthia Ménard

**Affiliations:** 1 Radiation Oncology Branch, National Cancer Institute, NIH-DHHS, Bldg 10, CRC Rm B2-3561, 9000 Rockville Pike, Bethesda, MD, 20892, USA; 2 Radiation Medicine Program, Princess Margaret Hospital, University Health Network, University of Toronto, 5th Floor, 610 University Avenue Toronto, Ontario, M5G 2M9, Canada

## Abstract

**Purpose:**

To report early observation of transient PSA elevations on this pilot study of external beam radiation therapy and magnetic resonance imaging (MRI) guided high dose rate (HDR) brachytherapy boost.

**Materials and methods:**

Eleven patients with intermediate-risk and high-risk localized prostate cancer received MRI guided HDR brachytherapy (10.5 Gy each fraction) before and after a course of external beam radiotherapy (46 Gy). Two patients continued on hormones during follow-up and were censored for this analysis. Four patients discontinued hormone therapy after RT. Five patients did not receive hormones. PSA bounce is defined as a rise in PSA values with a subsequent fall below the nadir value or to below 20% of the maximum PSA level. Six previously published definitions of biochemical failure to distinguish true failure from were tested: definition 1, rise >0.2 ng/mL; definition 2, rise >0.4 ng/mL; definition 3, rise >35% of previous value; definition 4, ASTRO defined guidelines, definition 5 nadir + 2 ng/ml, and definition 6, nadir + 3 ng/ml.

**Results:**

Median follow-up was 24 months (range 18–36 mo). During follow-up, the incidence of transient PSA elevation was: 55% for definition 1, 44% for definition 2, 55% for definition 3, 33% for definition 4, 11% for definition 5, and 11% for definition 6.

**Conclusion:**

We observed a substantial incidence of transient elevations in PSA following combined external beam radiation and HDR brachytherapy for prostate cancer. Such elevations seem to be self-limited and should not trigger initiation of salvage therapies. No definition of failure was completely predictive.

## Background

There are over 200,000 new cases and nearly 30,000 deaths each year from prostate cancer [1]. External beam radiation therapy (EBRT) and/or brachytherapy are mainstays of local therapy. Low dose rate (LDR) brachytherapy, with permanently implanted radioactive seeds, [2-6] and HDR brachytherapy, with temporarily implanted catheters, has been used to treat prostate cancer [7-10].

Prostate-specific antigen (PSA) is a sensitive measure of treatment outcome after radiotherapy (RT) for prostate cancer[11]. When RT is successful, the PSA level falls. If RT fails, the PSA increases over time. Independent of treatment, there is some natural variation in PSA levels. Given this variability, different definitions of failure have been suggested [12-15]. Though no definition is definitively superior, the ASTRO definition of failure (3 consecutive rises in PSA over the last 9 months) has been used in many large prostate cancer trials. After failure, patients may consider salvage therapy, including additional local therapies or hormone therapy.

Regardless of the definition, elevations in the PSA that rise and subsequently fall without treatment make it difficult to distinguish an actual failure from a transient and self-limited elevation. Such transient elevations, or benign 'PSA bounces', after EBRT and/or LDR brachytherapy have been described[13,14,16-23]. However, there is limited data on this phenomena following HDR brachytherapy[24].

This analysis describes early observations of the incidence of transient PSA elevation following external beam and HDR brachytherapy for prostate cancer.

## Methods

### Eligibility and accrual

Patients with intermediate- and high-risk localized prostate cancer were eligible to enroll if their disease profile included either Gleason score >6, or clinical stage greater than T2a (American Joint Committee on Cancer, 2002 edition), or prostate-specific antigen (PSA) level of ≥10 ng/mL, with no evidence of distant metastatic disease. Patient characteristics are shown in Table 1. One patient without a bounce had a Gleason score of 6. One patient in the bounce group had a Gleason score of 8. All others had a Gleason score of 7. Staging investigations included PSA measurement, complete blood count, digital rectal examination, histopathologic review, diagnostic endorectal coil MRI of the prostate, and bone scan in those with high-risk disease. Patients unsuitable for general anesthesia or MRI were excluded, as were patients who had undergone transurethral resection of the prostate (TURP) in the preceding 6 months, who had a large TURP defect, or had significant urinary symptoms as reflected by a high (>18) International Prostate Symptom Score. All eligible patients underwent preliminary MRI in the treatment position before enrollment to confirm adequate perineal access and the absence of pubic arch interference. Adjuvant hormonal or experimental PSA vaccine therapy was permitted at the discretion of the treating physician. All eligible prostate cancer patients who were evaluated for radiation therapy at the National Cancer Institute were informed about this study. Prior to enrollment, all patients provided written, informed consent in this IRB approved protocol.

**Table 1:** Patient and Treatment Characteristics

	Patients Without PSA Bounce (n = 4)	Patients With PSA Bounce (n = 5)	P Value
Median Age (years)	67	62	NS
			
Largest Field Treated			
Whole Pelvis Treated	0%	20%	NS
Prostate and Seminal Vesicles	75%	40%	NS
Prostate Only	25%	40%	NS
			
Concurrent Hormone Therapy	25%	60%	NS
T2b and greater tumor stage	0%	20%	NS
PSA less than 10	75%	80%	NS

### MRI guided HDR brachytherapy

The MRI guided HDR brachytherapy technique has been previously described[25,26]. Briefly, dwell time optimization was performed to achieve the following dosimetric parameters: target percentage of volume receiving 100% of prescribed minimal peripheral dose (V100) >90%, urethral V150 <2% and V125 <20%, and rectal V75 <2%. If the above-defined dosimetric parameters were achieved, a dose of 1050 cGy was prescribed to the 100% isodose. If they were not achieved, but a urethral V150 <2%, and rectal V75 <5% were obtained, 950 cGy was prescribed to the 100% isodose line.

### External Beam Radiation Therapy

Computed tomography simulation was performed 1 day after the first brachytherapy procedure with the patient in the supine position. The patient was instructed to void before simulation. CT images of the pelvis were obtained, and treatment planning for EBRT was performed. For those patients with high-risk localized disease (Gleason score ≥8, PSA ≥20 ng/mL, or Stage T3a or greater disease), the CTV included the prostate gland, seminal vesicles, and regional pelvic lymph nodes at risk. For all other patients with intermediate-risk localized disease, the CTV included the prostate gland and seminal vesicles. A planning target volume was obtained by adding a margin of 1.5 cm to the CTV. A dose of 4600 cGy was prescribed to the 100% isodose in 23 fractions, 200 cGy/d. During the last week of EBRT, or the week after EBRT, a second brachytherapy fraction was delivered in the same fashion.

### Hormone therapy

Hormonal therapy (with leuprolide acetate injection for 2 to 7 months following 2 weeks of oral biclutamide) was permitted during the trial at the discretion of the patient and treating physician and administered in a neoadjuvant, concurrent, and adjuvant fashion. For the purposes of this analysis, the 2 patients who received greater than 7 months of adjuvant hormone therapy were excluded. Five patients received no hormone therapy and 4 patients received neo-adjuvant and concurrent hormonal therapy, which was discontinued after RT.

### Definition of PSA bounce

Prostate-specific antigen (PSA) bounce was defined by a rise PSA values with a subsequent fall to levels less than the nadir or below 20% of the level of the maximum post-treatment PSA rise. The ability of 6 previously published definitions of biochemical failure to distinguish true failure from bounce were tested: definition 1, rise >0.2 ng/mL; definition 2, rise >0.4 ng/mL; definition 3, rise >35% of previous value; definition 4, ASTRO, definition 5 nadir + 2 ng/ml; and definition 6, nadir + 3 ng/ml.

### On treatment and follow-up evaluations

Patients were seen by a physician weekly while on treatment. Upon completion of therapy, follow-up visits occurred at 6 weeks, 3 months, 6 months, then every 6 months until 3 years, then annually until 5 years. The Radiation Therapy Oncology Group toxicity grades were documented at each follow-up visit. Patients with rising PSA were given the option of having monthly PSA tests.

### Statistical analysis

Summary statistics, such as sample proportions, means, and median values were used to describe the patient characteristics. A two-sided Fisher's exact test was used for comparing proportions across groups. A Wilcoxon rank sum test was used to compare medians across groups for continuous variables. All analyses were performed with MATLAB software (The Mathworks Inc, Natick, MA, USA).

## Results

**Figure 1:**
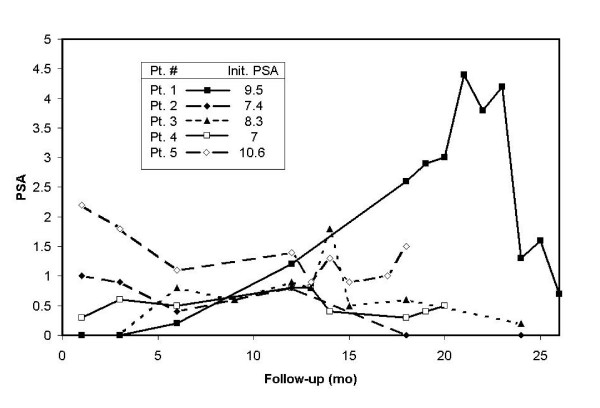
Transient PSA Elevations Following HDR Brachytherapy and External Beam Radiation. Pt = Patient. Init. PSA = Initial PSA. Patients 1, 3, and 4 were received hormone therapy for 7, 6 and 2 months respectively.

Median follow-up was 24 months (range 18–36 mo). During follow-up, 5 of 9 (56%) patients experienced a rise in their PSA which subsequently fell below nadir levels or to below 20% of the maximum PSA level. The first transient PSA rise occurred at 6 months in 2 patients and 12 months in the remaining 3 patients. The actual PSA values of all patients' experiencing a transient PSA elevation is plotted in Figure 1.

The incidence false positive "failures" due to such transient PSA elevation was: 56% for definition 1, 44% for definition 2, 55% for definition 3, 33% for definition 4, 11% for definition 5, and 11% for definition 6.

For the HDR brachytherapy implants, the following dosimetric parameters were evaluated: V100, V150, V200, urethra V100, urethra V125, prostate volume, number of catheters and catheter density. There were no significant differences between the group of patients who did and not experience a definition 1 transient PSA elevation.

## Discussion

This is the first paper to describe the incidence, amount, and duration of transient PSA elevations which subsequently fall without further treatment in patients treated with HDR brachytherapy and EBRT, with or without hormonal therapy. Multiple definitions of biochemical failure were used and all produced some false positives. The ASTRO definition of biochemical failure, one of the most common, had a 33% false positive rate.

Demanes et al, performed a review of 209 patients, without prior androgen suppression, treated with HDR-BT plus EBRT. Using a definition of failure with endpoints of local failure (determined by DRE or positive biopsy >2 years after treatment associated with PSA progression), distant failure, hormonal therapy, or a post-treatment PSA level >25 ng/mL, the authors reported 10 of 209 patients (4.8%) had false positive diagnoses of failure using the ASTRO definition. As in this study, Demanes et al found that the nadir plus 2 ng/ml definition correlated better with actual clinical outcome than the ASTRO definition. However, the authors did not further describe the nature of the amount or duration of PSA rise [24].

Rising PSA values after radiation therapy are worrisome for both patients and treating physicians. There is no clear evidence that very early initiation of salvage hormone therapy improves survival [27]. However, survival is better when hormones are initiated prior to development of distant metastases or when PSA is less than 20 [28]. Thus, there is an understandable desire to initiate salvage hormone therapy quickly prior to further potential advancement of disease.

Transient PSA elevations, which resolve without therapy, further complicate the decision to initiate salvage hormone therapy. Such transient elevations, or benign PSA bounces, after EBRT and/or LDR brachytherapy have been described [13,14,16-23]. The prognostic significance of PSA bounces following EBRT remains unclear [29,30]. PSA bounces do not appear to negatively impact long term outcome following LDR brachytherapy [13,19].

In a cohort of patients treated with LDR brachytherapy, using definition 1, Ciezki et al reported a 46% incidence of PSA bounce with a median time to occurrence of 15 months [13]. Using bounce definition 1, we found a similar 55% incidence but all patients had experienced bounce by 12 months. Therefore, time to bounce following HDR may be faster than following LDR.

The present study is the first to report the details of transient PSA rises in a population treated with HDR brachytherapy and EBRT. The optimal PSA-based definition to predict ultimate failure remains elusive necessitating evaluation of new definitions. Such evaluations will be facilitated if, as done here, authors graphically report the duration and magnitude of the PSA elevation.

Some patients in our study also received limited duration androgen deprivation therapy which is known to produce a high incidence of PSA bounce[14]. The small size of the present cohort combined with brief follow-up limits our ability to interrogate the causes and consequences of this PSA bounce.

We hope that our data will encourage reviews of larger databases of patients treated with HDR brachytherapy which will illuminate more optimal management thereby reducing both patient and physician anxiety.

## Competing interests

The author(s) declare that they have no competing interests.
